# The hindgut microbiota of praying mantids is highly variable and includes both prey-associated and host-specific microbes

**DOI:** 10.1371/journal.pone.0208917

**Published:** 2018-12-11

**Authors:** Kara A. Tinker, Elizabeth A. Ottesen

**Affiliations:** Department of Microbiology, University of Georgia, Athens, Georgia, United States of America; University of North Carolina at Chapel Hill, UNITED STATES

## Abstract

Praying mantids are predators that consume a wide variety of insects. While the gut microbiome of carnivorous mammals is distinct from that of omnivores and herbivores, the role of the gut microbiome among predatory insects is relatively understudied. Praying mantids are the closest known relatives to termites and cockroaches, which are known for their diverse gut microbiota. However, little is known about the mantid gut microbiota or their importance to host health. In this work, we report the results of a 16S rRNA gene-based study of gut microbiome composition in adults and late-instar larvae of three mantid species. We found that the praying mantis gut microbiome exhibits substantial variation in bacterial diversity and community composition. The hindgut of praying mantids were often dominated by microbes that are present in low abundance or not found in the guts of their insect prey. Future studies will explore the role of these microbes in the digestion of the dietary substrates and/or the degradation of toxins produced by their insect prey.

## Introduction

The gut microbiota of insects and other animals is known to play key roles in shaping host health. Gut microbes have been shown to assist in digestion and nutrient absorption, provide a layer of protection against opportunistic pathogens, and can metabolize certain toxins and/or environmental pollutants [1–3]. The gut microbiomes of many insect species have been studied extensively [2, 4–9], however, little work has been done that explicitly examines the gut microbiome of mantids (Dictyoptera: Mantodea). This order is of interest for several reasons. First, relatively little is currently known about the gut microbiota of insect predators. In mammals, the gut microbiome of carnivores is distinct from that of omnivores and herbivores [10], and diet has been suggested to influence gut microbiota diversity across insect species [11]. Second, as predators that often consume whole prey, mantids represent an opportunity to investigate the survival and retention of prey-associated gut microbes within the guts of predators. While the survival and retention of food-associated microbes in the gut has been discussed in many contexts, prey gut-associated microbes may represent a special case given their adaptation to the insect gut environment. Tiede et al. [12] recently found that lady beetles that were fed more diverse prey hosted increased gut microbiome diversity. However, they did not address the extent to which this increased diversity was associated with prey-derived microbes. Third, as frequent insect predators, mantids are potentially vulnerable to toxins produced by their prey [13–18]. As the gut microbiota of other insects has been implicated in degradation of diverse toxins and pesticides [3, 13, 15, 19–22], the mantid gut microbiota may contribute to host defenses against these prey-associated toxins. Finally, mantids are the closest known relatives to cockroaches and termites (Dictyoptera: Blattodea) [23]. Members of the Blattodea order are known for their remarkable gut symbioses [1, 5, 6, 24–30]. By studying the gut microbiota of praying mantids, we hope to gain insight into the origins of these symbioses.

Praying mantids are sympatric general predators that typically hunt based on prey size. Praying mantids primarily subsist on a diet of small insects including fruit flies, crickets, and caterpillars, although adult praying mantids are known to consume small birds or small reptiles [16, 31–34]. Common species, such as the Chinese mantis (*Tenodera sinensis)*, have been used to study and model predator behavior [35–40]. Recent work has begun to characterize the metabolic rates of praying mantises [41]. However, to our knowledge, there has been no in-depth study of praying mantis nutritional requirements. Emerging work suggests that mantids do not uniformly consume available insect prey [14, 16–18, 33, 42]. Praying mantids exhibit ontogenetic dietary patterns, occupying different trophic niches throughout their life cycle [33, 42]. Rather than hunting the most abundant prey, they target insect species with specific tissue nutrient profiles [33, 42]. As frequent predators of insects, mantids are vulnerable to challenge by toxic compounds produced by their insect prey to deter predation [13, 15]. Several mantid species are known to gut the body of certain insect prey to avoid specific toxins, such as cardenolide or atropine [14, 16–18]. These selective behaviors may extend beyond predator-prey interactions, as praying mantids are known to ingest a high-protein herbivorous diet of pollen when there is no available prey [43]. Chinese mantids that consume an omnivorous diet are more fit than their strict carnivore counterparts, with larger body mass, higher fecundity, and increased offspring survival [43].

Published work on the gut microbiome has demonstrated that most insects host simple gut microbial communities that are highly variable among individuals of the same species [2]. Insects found in the Blattodea order are among the exceptions: cockroaches host a complex gut microbiome composed of hundreds of unique microbial species that is highly stable between individuals [24–28, 30] while termites are well known for their obligate symbioses with a complex gut microbial community [1, 5, 6, 29]. As the closest known relative to cockroaches and termites (Dictyoptera: Blattodea), we sought to examine the gut microbiota of praying mantids (Dictyoptera: Mantodea) to explore whether their gut microbiota provide insight into the evolutionary origin of this symbiosis. These insects are closely related, however they exhibit drastically different lifestyles. Praying mantids are solitary predators that typically survive on a carnivorous diet [44, 45]. In contrast, cockroaches are gregarious and consume an omnivorous diet [24–28, 30] while termites are eusocial and consume a herbivious diet [1, 5, 6, 29]. Although the exact effects of dietary lifestyle and social interaction on the gut microbiome are still unclear, these environmental factors have been linked with significant changes in gut microbiome structure and diversity in multiple animal species, including insects [2, 10, 11, 27, 46–50].

A 2014 study used 454 pyrosequencing of 16S rRNA genes to complete a survey of the gut microbiome from 305 insects across multiple orders [11]. This survey included 6 mixed-age praying mantids (3 *T*. *sinensis* and 3 *Tenodera angustipennis)*, with an average depth of 552 sequences per sample [11]. Due to the high number of total insect samples and limited sequencing depth, Yun et al.’s analysis focused on broad trends across insect order, diet, age, and/or environmental habitat. Yun et al. reported that the gut microbiota of most insects, including those in the Mantodea and Blattodea orders, were primarily composed of bacteria from the Proteobacteria and Firmicutes phyla [11]. No other information was reported on the Mantodea gut microbiome and, to our knowledge, no other study has been conducted which examines the structure of the praying mantis gut microbiome. We aimed to fill this knowledge gap by completing a survey of the hindgut microbiota from 15 individual praying mantids across three species using 16S rRNA sequencing. Praying mantids were fed a uniform diet of cockroaches, allowing us to 1) characterize the praying mantis gut microbiome and 2) determine if the praying mantis gut microbiome is composed solely of members of the prey microbiome or if it hosts a unique gut microbiota populated by mantid-associated bacterial lineages.

## Methods

### Insects and sample collection

30 fourth-instar praying mantis nymphs were purchased from Bugs in Cyberspace (Stafford, Oregon) where they were reared on a diet of *Drosophilia*. Three species, with ten nymphs per species, were represented: *T*. *sinensis*, *Hierodula venosa*, and *Deroplatys lobata*. Nymphs were kept isolated in plastic cups, with a pipe cleaner for perching and a cotton ball damp with water. Nymphs were initially fed daily and raised on a mixed diet of *Drosophilia melanogaster*, *Drosophilia hydei*, and *Periplaneta americana* nymphs. After several molts, surviving praying mantids were exclusively fed *P*. *americana* every 1–2 days until their sacrifice. In nearly all cases, mantids were observed to consume prey insects in their entirety, including the gut and gut contents. All insects were offered prey within 24 hours of sacrifice.

The initial aim of the work was to sacrifice healthy adult mantids at one week past their final molt. Given high rates of insect loss, particularly following the final adult molt, some adults and nymphs were sacrificed early when their overall condition and activity levels suggested that they were unlikely to survive to the target age (Tables 1 and S1). Mantids classified as being in excellent condition were dissected several days after their final molt, while those classified as poor were adults or nymphs that unsuccessfully molted and were unlikely to survive. Very poor condition refers to mantids that unsuccessfully molted and were visibly infected with parasitic worms.

**Table 1:** Metadata for praying mantids.

Sample ID	Species	Age (Adult or Nymph)	Condition (Excellent/Poor/Very Poor)
W1	*Tenodera sinensis*	Adult	Excellent
W2	*Tenodera sinensis*	Adult	Excellent
W3	*Tenodera sinensis*	Nymph	Poor
W4	*Tenodera sinensis*	Nymph	Poor
W5	*Tenodera sinensis*	Nymph	Poor
X1	*Hierodula venosa*	Adult	Excellent
X2	*Hierodula venosa*	Nymph	Poor
X3	*Hierodula venosa*	Nymph	Poor
X4	*Hierodula venosa*	Nymph	Poor
X5	*Hierodula venosa*	Nymph	Poor
X6	*Hierodula venosa*	Nymph	Poor
X7	*Hierodula venosa*	Nymph	Very Poor
X8	*Hierodula venosa*	Nymph	Very Poor
Y1	*Deroplatys lobata*	Nymph	Poor
Y2	*Deroplatys lobata*	Nymph	Poor

*P*. *americana* were provided by the University of Georgia’s entomology department. They are maintained in mixed-age, mixed-sex colonies in aquarium tanks on a diet of dog food (Pet Pride Chunk Style Complete Nutrition Dog Food [Pet Pride], composed of 21% protein, 9% fat, and 4% fiber) *ad libitum*. Healthy adult cockroaches were selected at random from the tank for dissection, and all were in excellent condition at time of sacrifice (S1 Table).

Praying mantids and cockroaches were placed in CO_2_ chambers or on ice in sterile culture plates until sufficiently torpid (Tables 1 and S1). The insects were then dissected and the whole gut was removed. The whole gut was rinsed with 1XPBS diluted from 20X stock at pH 7.5 (Amresco, Solon, OH), and visible debris was removed with forceps. The whole gut was placed on a sterile surface before separating the hindgut with a flame-sterilized scalpel. After separation, the hindgut was placed in 1XTE Buffer and stored -80°C.

### DNA extraction

A modified version of the EZNA Bacteria Kit (Omega Biotek, Norcross, GA) was used to extract microbial DNA from stored hindgut samples. After thawing on ice, samples were pulverized with a sterile microcentrifuge pestle. Pulverized samples were centrifuged for 10 min at 5,000 g, the supernatant was discarded, and the pellet was resuspended in 100 μL of 1XTE Buffer. 10 μL lysozyme (as supplied by kit) was added to the resuspended sample before incubation at 37°C for 30 min. After incubation, approximately 25 mg of glass beads (as supplied by the kit) was added to each sample. Samples were bead beaten for 5 min at 3,000 rpm using a vortex mixer with a horizontal adapter. Next, 100 μL BTL buffer and 20 μL proteinase K solution (as supplied by the kit) were added to each sample before incubation at 55°C while shaking at 600 rpm for 1 h. After incubation, the manufacturer’s protocol (June 2014 version) was followed beginning at step 11. The final DNA concentrations (ranging from 40 to 800 ng/μL) and A260/A280 were measured using a NanoDrop Lite spectrophotometer (Thermo Scientific, Wilmington, DE).

### Library preparation and sequencing

A previously described two-step PCR method was used to prepare the amplicon library for sequencing [30]. During the initial reaction the V4 region of the 16S rRNA gene was amplified with the 515F (GTGCCAGCMGCCGCGGTAA) and 806R (GGACTACHVGGGTWTCTAAT) primers. The initial reaction contained 1X Q5 buffer (New England Biolabs [NEB], Ipswitch, MA), 200 μM dNTPS, 0.5 μM 515F, 0.5 μM 806R, 2 ng template DNA, and 0.02 U/μL Q5 Hot Start High-Fidelity DNA polymerase (NEB) for a total reaction volume of 10 μL. PCR conditions were 98°C for 30 s for denaturation followed by 15 cycles at 98°C for 10 s, 52°C for 30 s, and 72°C for 30 s. The final extension step was 72°C for 2 min.

Immediately after the final extension, 9 μL of the initial reaction product was reamplified using barcoded 515F and 806R primers [30] (S2 Table). The initial reaction product was added to the second reaction, which contained 1X Q5 buffer (NEB), 200 μM dNTPS, 0.5 μM 515F, 0.5 μM 806R, and 0.02 U/μL Hot Start High-Fidelity DNA polymerase (NEB) at a volume of 21 μL. PCR conditions were 98°C for 30 s for initial denaturation; 4 cycles at 98°C for 10 s, 52°C for 10 s, and 72°C for 30 s; 6 cycles at 98°C for 10 s and 72°C for 1 min; and the final extension step at 72°C for 2 min.

Amplicons were visualized on a 2% w/v agarose gel. PCR for failed samples was redone with an additional 5 cycles during the initial reaction (S1 Table). Amplicons were purified using the EZ Cycle Pure Kit (Omega Biotek) before quantification with a NanoDrop Lite spectrophotometer (Thermo Scientific). Samples were normalized and pooled to a concentration of 10 nM on the basis of a predicted total product size of ~400bp. The pooled library was submitted to the Georgia Genomics facility for sequencing (Illumina MiSeq 250x250 bp; Illumina, Inc., San Diego, CA).

### Data analysis

The Mothur software package was used to analyze 16S rRNA gene sequences generated during this experiment [51]. To allow for direct comparison, the guts of 15 adult laboratory cockroaches were prepared, sequenced, and analyzed in parallel with praying mantis gut microbiome data. A modified version of the Miseq standard operating protocol [52, 53] was followed. Sequences were assembled and screened to remove any that contained ambiguous bases or were longer than 275 base pairs. Remaining sequences were aligned to the Silva reference database (Release 128) [54–56]. After alignment, sequences were screened to remove any that contained homopolymers of 8 or more base pairs. Chimeras were identified via UCHIME and removed [57]. Remaining sequences were classified using the DicDB reference database (Version 3.0), a specialized Silva-based reference database that provides high resolution for gut microbiota associated with cockroaches and termites [58]. Any sequences that were unclassified or identified as chloroplasts, mitochondria, Eukaryota, or *Blattabacterium* (cockroach endosymbiont found in fat body cells) were removed. The remaining sequences were clustered using the OptiClust [59] method into OTUs based on 97% or greater sequence identity.

Data generated by Mothur was imported into R for further analysis [60]. We utilized the vegan package [61] to calculate Shannon diversity and Bray-Curtis dissimilarity values for our data. We also used vegan to complete non-metric multidimensional scaling (NMDS) analyses and permutational multivariate analysis of variances (PERMANOVA) on these calculated metrics.

### Accession numbers

The sequences generated from this experiment were submitted to the NCBI Sequence Read Archive and are available under BioProject accession numbers SRP132948 and SRP132487.

## Results

16S rRNA gene libraries were prepared and sequenced from hindguts of praying mantids fed a diet of laboratory-raised cockroaches and from hindguts of laboratory-raised prey cockroaches. This 16S rRNA library resulted in a total of 1,426,892 raw sequences, with 939,355 remaining after quality control filtering (S1 Table).

**Fig 1:**
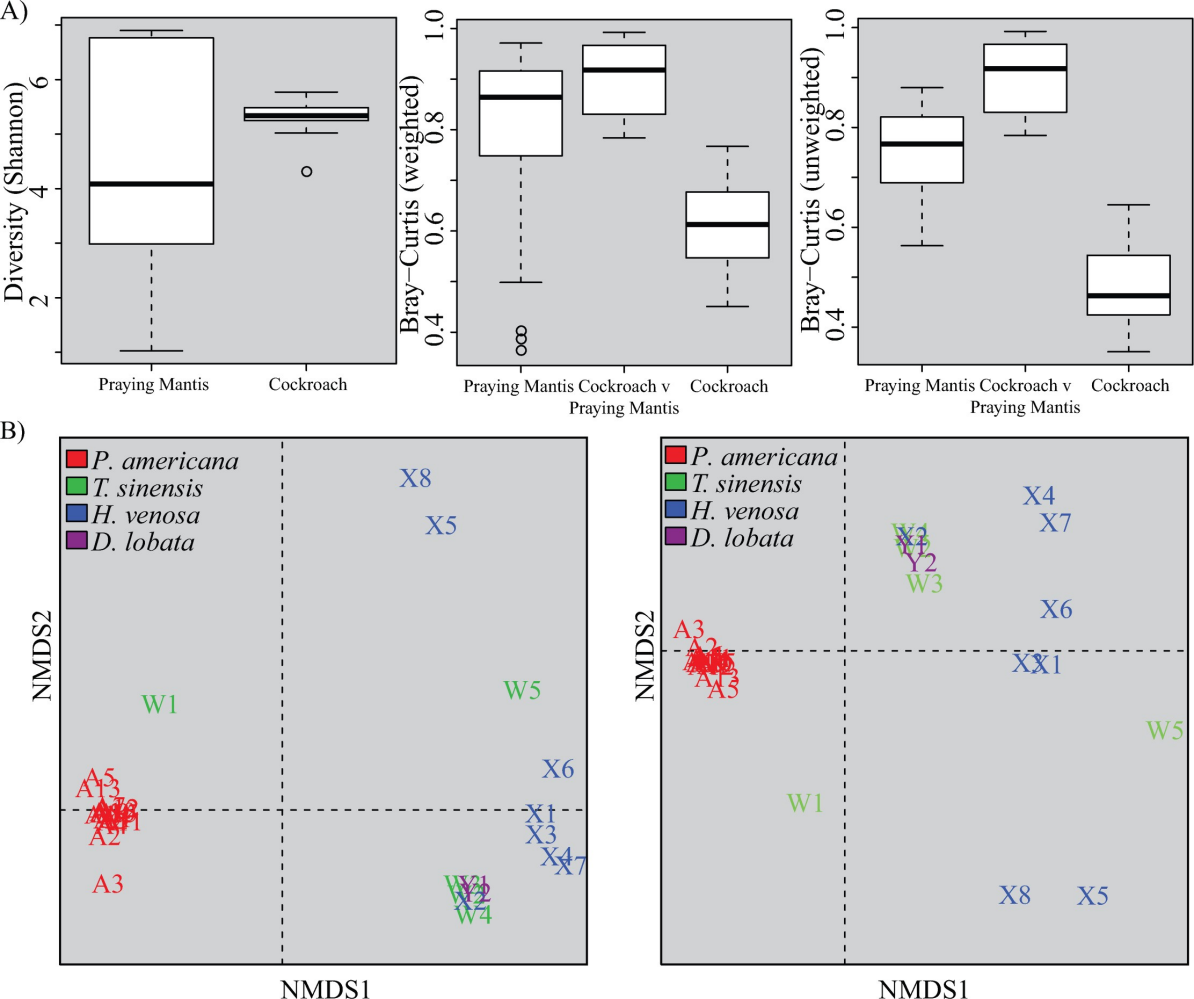
Alpha and beta diversities among praying mantids and cockroaches. A) Boxplots show Shannon diversity indices (left), and weighted (middle) and unweighted (right) Bray-Curtis dissimilarities among praying mantid and cockroach gut microbial communities at 97% sequence identity. The boxplots with Bray-Curtis metrics also display the dissimilarity between praying mantid and cockroach gut microbial communities. Libraries were resampled to a depth of the sample with the fewest sequences (3901). For each group, the bars delineate the median, the hinges represent the lower and upper quartiles, the whiskers extend to the lesser of the most extreme value or 1.5 times the interquartile range, and outliers are plotted, if present. B) Nonmetric multidimensional scaling (NMDS) plots of insects constructed with weighted (left) and unweighted (right) metrics. Libraries were resampled to a depth of the sample with the fewest sequences (3901). Constructed plots had stress values of 0.044 (weighted) and 0.046 (unweighted). Points are color coded by insect species and were left in text format, such that the text at each point corresponds to the metadata provided in Tables 1 and S1.

Praying mantids were found to harbor diverse gut microbiota. As a whole, these insects exhibited lower alpha diversity and higher beta diversity in gut microbiome composition than their cockroach prey. Although the mean alpha diversity observed in mantids (Shannon Index: 4.4) was lower than in cockroaches (Shannon Index: 6.7), there was no significant difference in the means (T-test, p > 0.05). However, variance in alpha diversity was significantly higher (F-test, p < 0.001 (Figs 1A and S1) such that an individual praying mantis may possess a gut microbiota that is equally or more diverse than the typical cockroach gut microbiota, with Shannon indices ranging from 4.2–5.8 for cockroaches and 1.0–6.9 for mantids.

Inter-individual variation among praying mantids was substantially higher than observed in cockroach prey by both weighted and unweighted beta diversity measures (Fig 1A and S1). Similarly, NMDS analyses show much tighter clustering of cockroach samples than mantids (Fig 1B). NMDS analyses also show clear separation between mantid and cockroach samples and PERMANOVA found significant differences in gut microbiome composition between mantids and their cockroach prey (R^2^ = 0.211, p-value < 0.001). In contrast, we did not find significant relationships between gut microbiome composition and praying mantis age or condition (p-value > 0.05).

Firmicutes, Proteobacteria, and Bacteriodetes were the predominant phyla present in the hindgut microbiota of all insects. All hindguts also contained a high proportion of microbes from other, less abundant phyla including Actinobacteria, Verrucomicrobia, and Tenericutes. Variability among individual insects was higher in praying mantids, with non-predominant phyla composing 3–40% of the gut microbiota in praying mantids and only 5–13% in cockroaches (S2 Fig).

**Fig 2:**
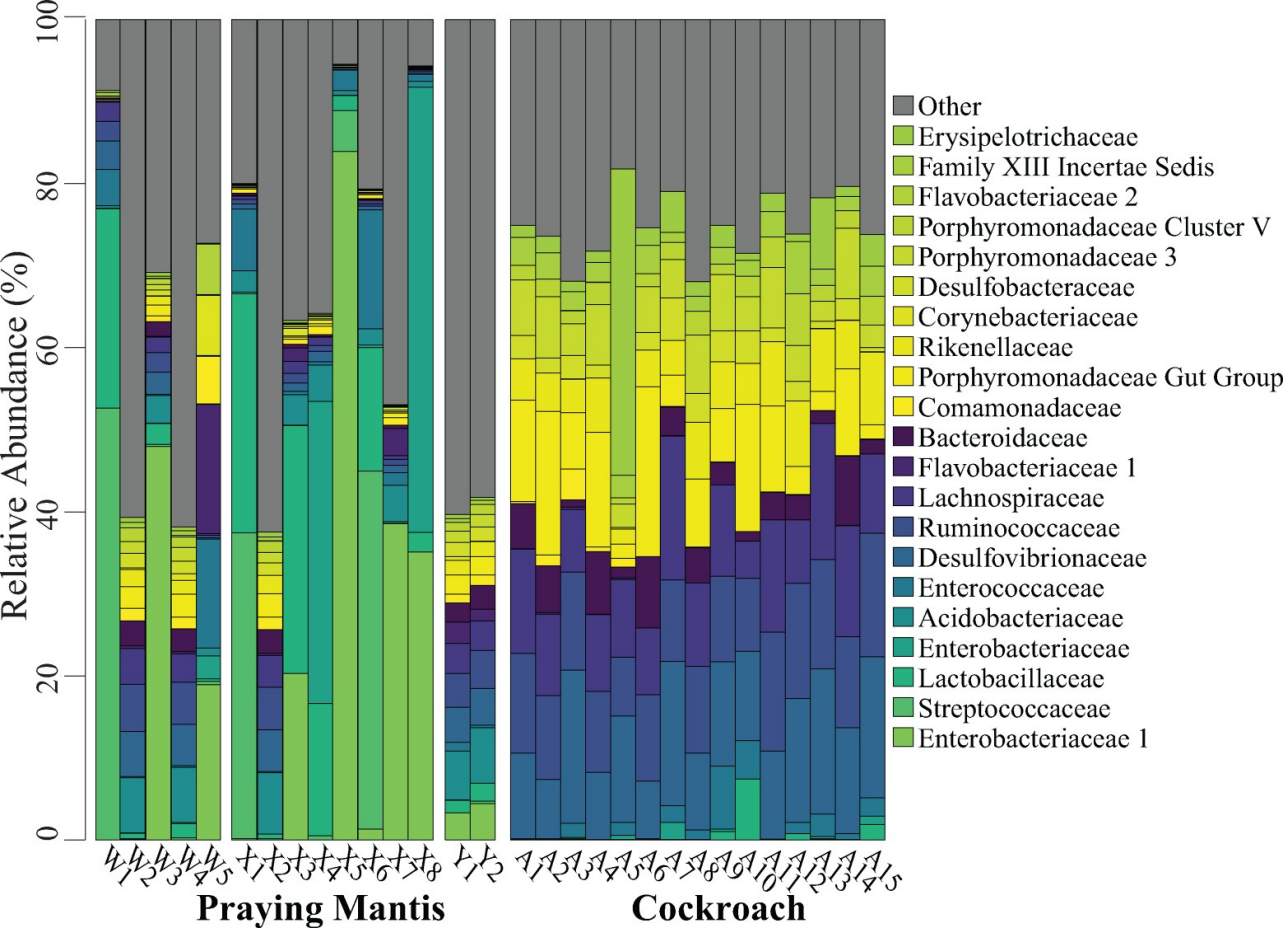
Relative abundance of microbial families in praying mantids and cockroaches. Each bar represents an individual insect gut. All families that represent ≥5% from any one sample are listed in the legend; less abundant families are grouped together under Other.

The Streptococcaceae, Lactobacillaceae, and Enterococcaceae families were prevalent among praying mantids, representing 75% of all Firmicutes found in praying mantis hindgut samples (Fig 2). In contrast, these families only composed 6.1% of all Firmicutes found in prey hindgut samples. Within the Proteobacteria phylum, Enterobacteriaceae were the most abundant family in the gut microbiota of praying mantids. Enterobacteriaceae represented 41.6% of all Proteobacteria found in praying mantid hindgut samples, but were not present in any of the cockroach samples (Fig 2).

While Enterobacteriaceae were found only within praying mantids, other bacterial lineages were common to both praying mantids and their cockroach prey. For example, the majority of Bacteriodetes in both insect orders are members of the Flavobacteriaceae 1, Bacteroidaceae, and Porphyromonadaceae gut group families (Fig 2). These families composed a total of 45.6% and 39.2% of all Bacteriodetes in the praying mantid and cockroach hindguts, respectively.

**Fig 3:**
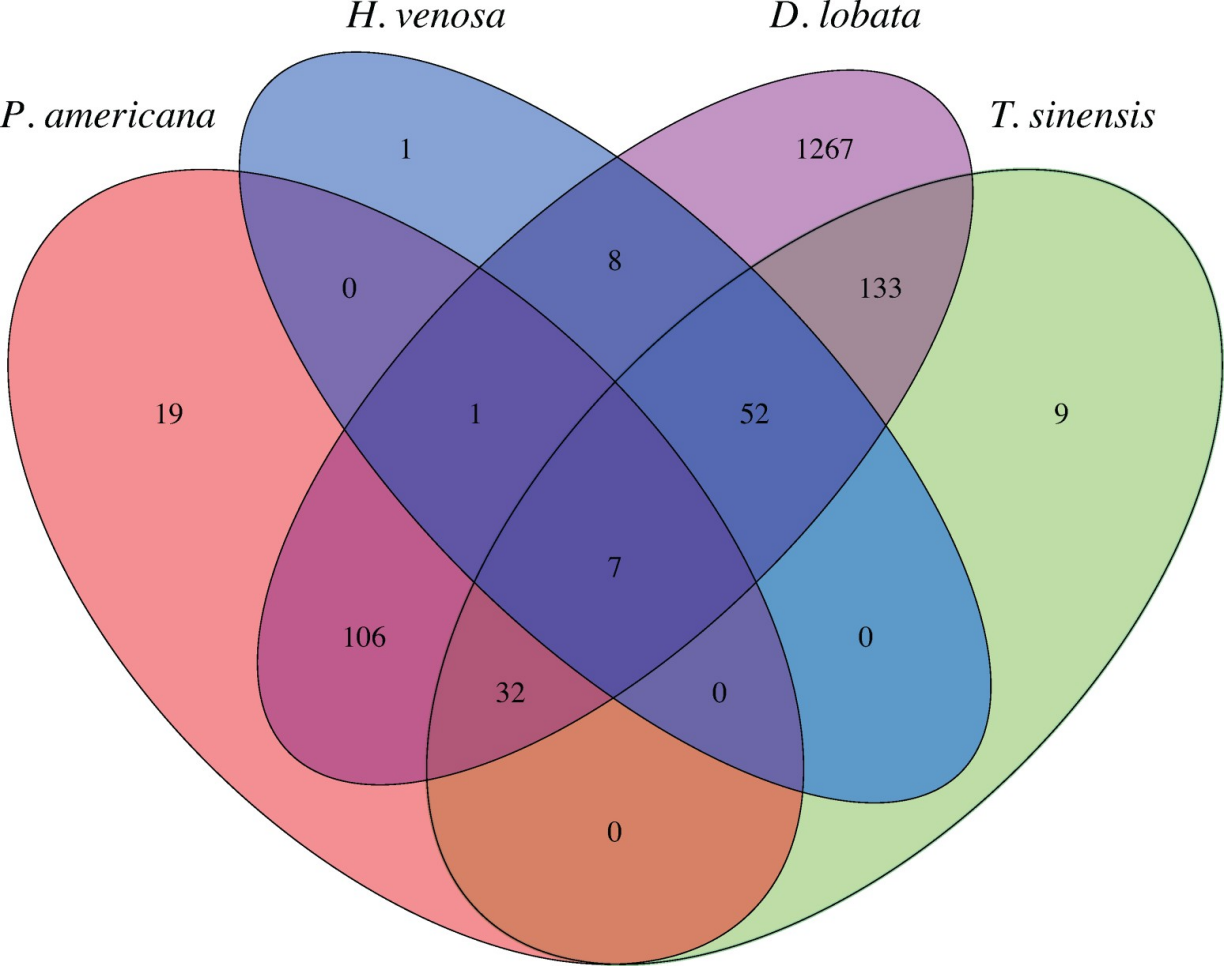
Venn diagram of number of core OTUs shared among all praying mantis and cockroach species. 7 OTUs were shared among all insect species: an *Enterococcus*; a cluster of Ruminococcaceae associated with the Blattodea order; Comamonadaceae; *Rhodoferax*; Desulfobacteraceae; *Bacteroides*; and a cluster of Porphyromonadaceae associated with termites (S3 Table). Analysis utilized un-resampled datasets. A list of OTUs shared among all insects is shown in S3 Table, all mantids in S4 Table, and all cockroaches in S5 Table. S3–S5 Tables also contain the relative abundance of these core OTUs across all samples, all mantids, and all cockroaches, respectively. OTUs shared among each species of praying mantid are found in S6–S8 Tables. This venn diagram was generated in R with the VennDiagram package [60, 62].

**Fig 4:**
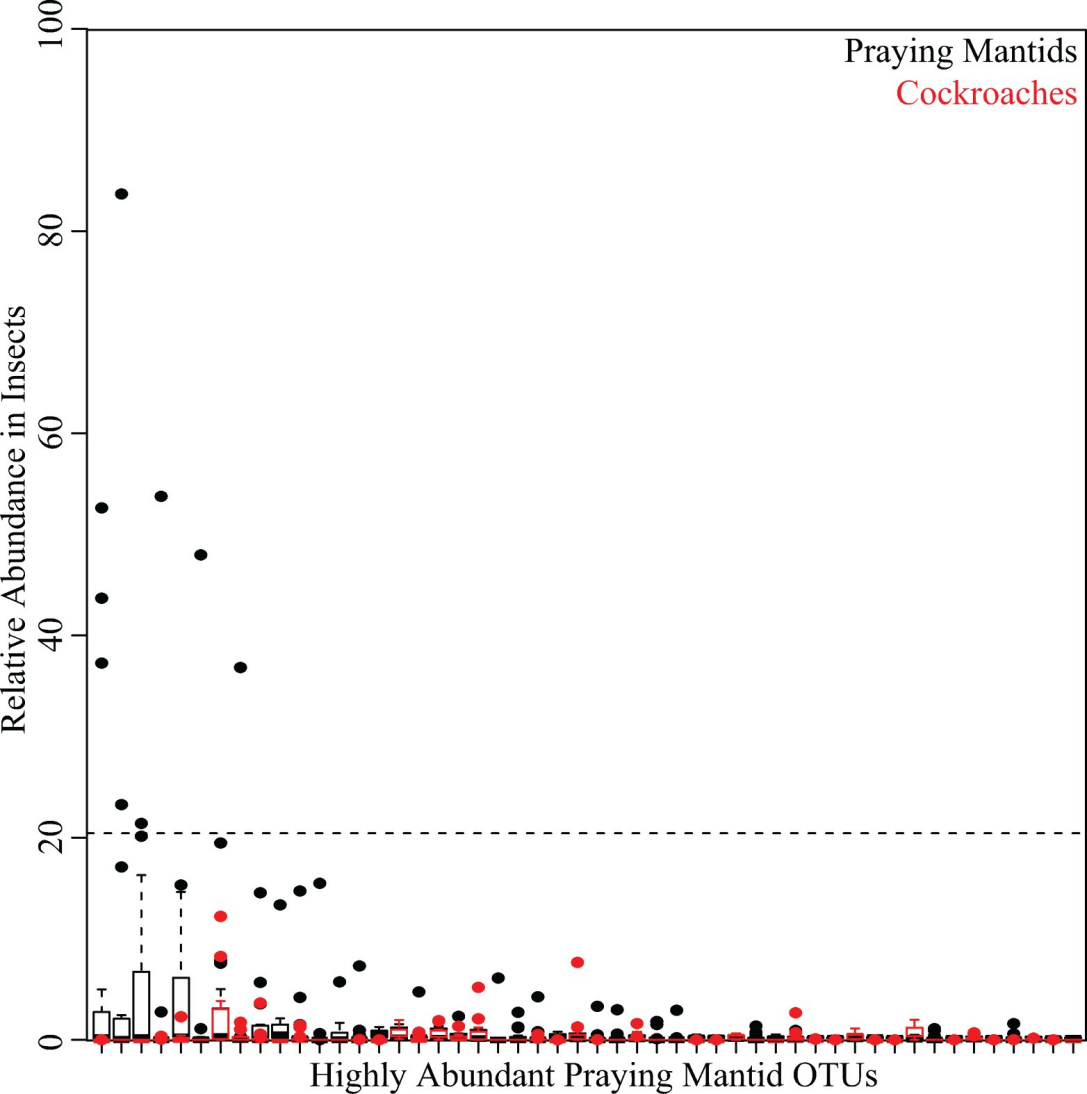
Relative abundance of the 50 most abundant praying mantis OTUs in insects. Two boxplots are shown at each OTU; they depict the relative abundance of that OTU among all praying mantids (black) and all cockroaches (red). For each boxplot, the bars delineate the median, the hinges represent the lower and upper quartiles, the whiskers extend to the lesser of the most extreme value or 1.5 times the interquartile range, and outliers are plotted, if present. There are 10 OTUs that were above 20% relative abundance within a single gut sample. These 10 OTUs were present in 9 unique mantid individuals (one mantid had two OTUs present at > 20%).

At the 97% OTU level, few lineages were shared between praying mantids and cockroaches. Only 7 OTUs were found in all insect microbiomes in the study, with 52 shared among all praying mantids (but not cockroaches) and 19 shared among all cockroaches (Fig 1A and S2–S7 Tables). The 52 OTUs shared among all mantids were found in low abundance within most mantids, with a median of <1% relative abundance for all OTUs. The most abundant OTUs found in praying mantids are either completely absent from all cockroaches or only found in a few cockroaches at low abundance (Fig 3). These highly abundant OTUs are not found at equal depth across all praying mantids. A closer examination reveals 10 OTUs which represent >20% relative abundance from the praying mantis individual in which they were present. These 10 OTUs are distributed among 9 unique praying mantis individuals, with only one praying mantis harboring two high abundance OTUs (Fig 4).

## Discussion

Our work suggests that praying mantids have a unique, host-associated gut microbiota that exhibits a distinct phylogenetic profile from the gut microbiota of prey and is dominated by lineages that are either not found or not abundant in cockroaches. This result is in contrast to emerging work on the caterpillar gut microbiome, which demonstrates some insects have no resident microbiota and instead harbor transient, diet-associated microbiota [63]. Potential sources of these microbes include vertical transmission, environmental contact, and/or inoculation from prey insects from early in life. While all mantids were fed cockroaches in the final weeks of the study, mantids were fed a combination of *D*. *melanogaster* and *D*. *hydei* until large enough to safely consume cockroach nymphs. It is also possible that unique microbes were acquired from nymphal cockroach prey, as only adult cockroaches were included in the comparison dataset. However, work by Carrasco et al. demonstrates that cockroach nymphs older than two weeks possess a gut microbiota that is highly similar to that of adult cockroaches [64].

Many of the predominant bacterial lineages found in mantid guts showed substantial variance in overall abundance across individuals. Due to the destructive sampling techniques used, it is impossible to say whether this variability is the result of inter-individual variability that is paired with intra-individual temporal stability or whether the observed variability is the result of frequent microbial blooms and temporal variability within the guts of individual mantids. While diet was held constant for these mantids, potential sources of variability may include time since last meal (which varied from immediately before sacrifice up to 24 hours before sacrifice) which may have implications for the loss rate of ‘transient’ prey-associated microbes. Variability in gut microbiota composition may also have resulted from variability in microbes introduced early in life, as *Drosophila sp*. (the nymphal diet of our mantids) are known to harbor extensive individual-to-individual variability in their gut microbiota [65]. In addition, given the poor health of some insects at sacrifice, we cannot rule out that some of these taxa may be opportunistic pathogens responding to dysbiotic events in the gut. However, while our sample size is limited, no significant differences in gut microbiome composition were identified between healthy adult mantids and individuals sacrificed early due to poor condition (PERMANOVA, p-value >0.05), suggesting the high variation in observed microbiome composition may be typical.

Although there was high variability in the gut microbiota among the sampled mantids, a core community of 52 OTUs was found in every mantid hindgut (S4 Table). The OTUs in this core community were found in low abundance among all mantids, with each OTU composing an average of <1% of the total relative abundance for each mantis. However, this core mantid community was nearly three times larger than the group of presumptive bacterial species shared only among all cockroaches sampled (19 OTUs). Many of these 52 OTUs include members of the Actidobacteria, Bacteroidetes, Firmicutes, Gemmatimonadetes, Nitrospirae, Planctomycetes, Proteobacteria, and Verrucomicrobia phyla. Several of these phyla were not found in any of the praying mantids sampled by Yun et al [11]. This is unsurprising due to the low sequencing depth of these samples, which averaged 552 sequences per mantid compared to our average of 31, 231 sequences per mantid [11]. These 52 core OTUs are unclassifiable at high taxonomic resolutions, with most unresolved past the order or family level. This makes it challenging to predict the functional roles of these taxa within the gut mantid gut microbiome. However, some of these core OTUs may provide assistance in neutralizing and/or degrading common environmental or prey-associated toxins. While cockroaches and *Drosophila* are not known to produce or carry toxins, in the wild mantids routinely prey on insects, such as caterpillars, that utilize alleochemical defense mechanisms [14, 16–18]. Emerging work demonstrates that the insect gut microbiome can assist with the degradation of toxic substances, such as the alleochemical cardenolide or the pesticide chlorpyrifos [3, 13, 15, 19–22]. Gut microbes associated with these activities include numerous *Lactobacillus* and *Enterococcus sp*., including *Lactobacillus lactis* and *Enterococcus faecalis* [3, 19]. OTUs assigned to *Lactobacillus* and *Enterococcus sp*. were present in the gut microbiota of multiple mantids and three of the 52 core mantid OTUs were *Lactobacillus sp*.

One goal of this work was to determine the extent to which the gut microbiome of praying mantids resemble those of other members of superorder Dictyoptera. Our results suggest that, unlike cockroaches and termites, the praying mantis possess a gut microbial community that is highly variable between individuals, with lower average alpha diversity and higher beta diversity than observed in cockroaches. This difference may be due to the predatory diet and/or solitary lifestyle of mantids. Previous work has suggested that omnivorous insects have more diverse gut microbiota than insects that were solely carnivorous or herbivorous, which might explain in part the higher alpha diversity observed in cockroaches [11]. Alternatively, the gregarious and/or social lifestyles of cockroaches and termites offer more opportunity for transfer of gut microbes between parents and offspring, and therefore more opportunity to establish stable and co-evolutionary relationships between host and gut microbiota [2].

In conclusion, this work suggests that mantids possess a diverse and highly variable gut community microbiota that is unique in composition from that of their prey. Overall, this gut microbiome is more typical of that observed in other insects than the highly diverse and stable microbiota found in other members of the superorder Dictyoptera. This work represents the first in-depth examination of the praying mantis gut microbiome and provides a foundation for future work on the origins of the predator gut microbiome and its role in host health and nutrition.

## Supporting information

S1 Table Sample metadata for all insects. (XLSX)

S2 Table Barcodes used in primers. (DOCX)

S1 Fig Alpha and beta diversities among praying mantids and cockroach species. Boxplots show Shannon diversity indicies (left), and weighted (middle) and unweighted (right) dissimilarities among praying mantid and cockroach gut microbial communities at 97% sequence identity. Libraries were resampled to a depth of the sample with the fewest sequences (3901). For each group, the bars delineate the means, the hinges represent the lower and upper quartiles, the whiskers extend to the most extreme values (which are no more than 1.5 times the interquartile range from the box), and outliers are plotted, if present.
(TIF)

S2 Fig Relative abundances of microbial phyla in praying mantids and cockroaches. Each bar represents an individual insect gut. All phyla that represent ≥1% of sequences from any one sample are listed in the barplot, all other phyla are grouped together under 'Other'.
(TIF)

S3 Table Core OTUs found in the gut microbiota of all insects and the relative abundance of these OTUs. (XLSX)

S4 Table Core OTUs found in the gut microbiota of all praying mantids and the relative abundance of these OTUs. (XLSX)

S5 Table Core OTUs found in the gut microbiota of all cockroaches and the relative abundance of these OTUs. (XLSX)

S6 Table Core OTUs found in the gut microbiota of all *T*. *sinensis*. (XLSX)

S7 Table Core OTUs found in the gut microbiota of all *H*. *venosa*. (XLSX)

S8 Table Core OTUs found in the gut microbiota of all *D*. *lobata*. (XLSX)
