# Supplementary material for: The hindgut microbiota of praying mantids is highly variable and includes both prey-associated and host-specific microbes
Source: PLoS One. 2018 Dec 11;13(12):e0208917. doi: 10.1371/journal.pone.0208917 (PMC6289422; doi:10.1371/journal.pone.0208917)
Supplement: S2 Table — (DOCX) [file pone.0208917.s002.docx]

**S2 Table. Barcodes used in primers**

| **Forward Barcode** | **Reverse Barcode** |
| --- | --- |
| 1. AACCAACC | 1. GTGTGTGT |
| 2. CCAACCAA | 2. AACGAACG |
| 3. GGTTGGTT | 3. TGTCTCAC |
| 4. TTGGTTGG | 4. CCAACGTA |
| 5. AGTCGACT | 5. CGTAGCAT |
| 6. CCATCCTA | 6. TTCGTTCG |
| 7. GTCAAGAG | 7. ACACAGTC |
| 8. TAGGTTGC | 8. GAGTCAGA |
| 9. AAGCAAGC | 9. CGATGGTT |
| 10. CGTTCGTT | 10. ATCGTTGG |
| 11. GCAAGCAA | 11. TAGCAACC |
| 12. TTCGTTCG | 12. GCTACCAA |
| 13. AGGTGAAC | 13. CACTGAGT |
| 14. CTACAGCA | 14. AGTGTCTG |
| 15. GACACTGT | 15. TCACAGAC |
| 16. TCTGTGTC | 16. GTGACTCA |

515F Primer: AATGATACGGCGACCACCGAGA TCTACAC XXXXXXXX TATGGTAATT CA GTGCCAGCMGCCGCGGTAA; 806R Primer: CAAGCAGAAGACGGCATACGAGAT XXXXXXXX AGCAGCTCCAG AC GGACTACHVGGGTWTCTAA
