# Supplementary figures and images for: The hindgut microbiota of praying mantids is highly variable and includes both prey-associated and host-specific microbes
Source: PLoS One. 2018 Dec 11;13(12):e0208917. doi: 10.1371/journal.pone.0208917 (PMC6289422; doi:10.1371/journal.pone.0208917)

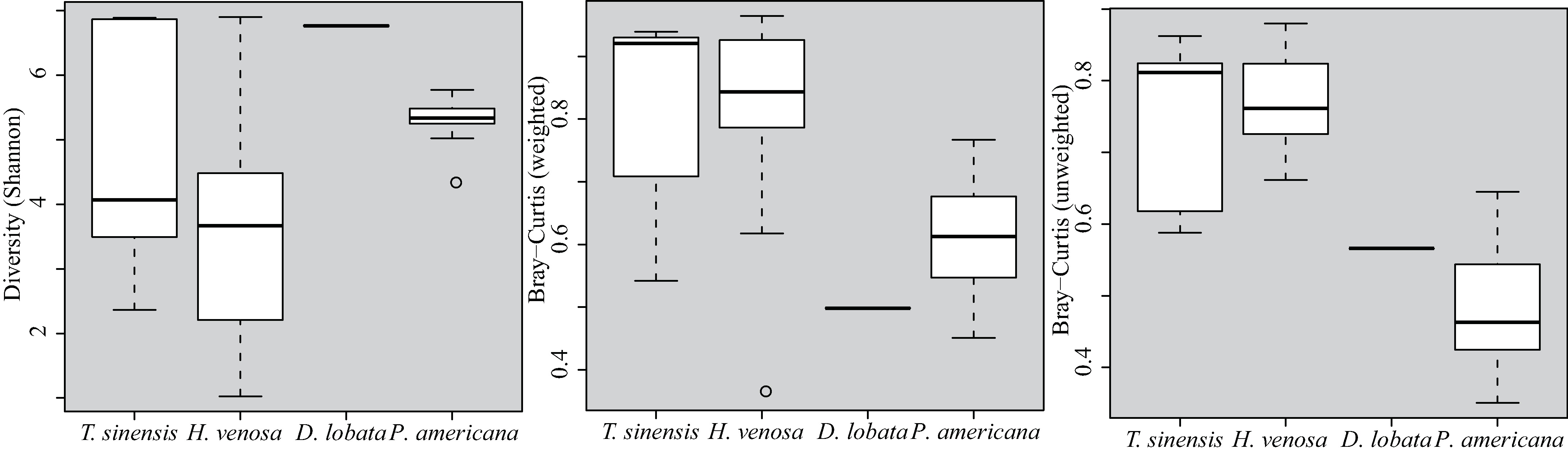

Supplement: S1 Fig — Boxplots show Shannon diversity indicies (left), and weighted (middle) and unweighted (right) dissimilarities among praying mantid and cockroach gut microbial communities at 97% sequence identity. Libraries were resampled to a depth of the sample with the fewest sequences (3901). For each group, the bars delineate the means, the hinges represent the lower and upper quartiles, the whiskers extend to the most extreme values (which are no more than 1.5 times the interquartile range from the box), and outliers are plotted, if present. (TIF) [file pone.0208917.s003.tif]

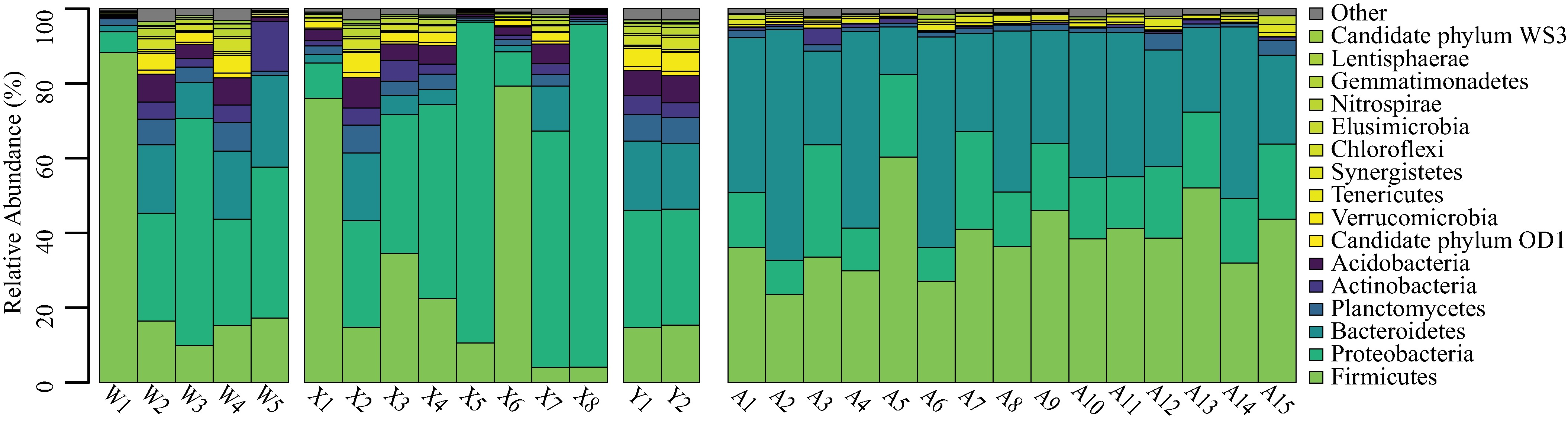

Supplement: S2 Fig — Each bar represents an individual insect gut. All phyla that represent ≥1% of sequences from any one sample are listed in the barplot, all other phyla are grouped together under 'Other'. (TIF) [file pone.0208917.s004.tif]
